# Understanding Diversity Ideologies From the Target's Perspective: A Review and Future Directions

**DOI:** 10.3389/fpsyg.2019.00282

**Published:** 2019-02-27

**Authors:** Seval Gündemir, Ashley E. Martin, Astrid C. Homan

**Affiliations:** ^1^Work and Organizational Psychology, University of Amsterdam Amsterdam, Netherlands; ^2^Stanford Graduate School of Business, Stanford University Stanford, CA, United States

**Keywords:** diversity, diversity management, organizational psychology, minority–majority, workplace equality

## Abstract

We present a review of the diversity ideologies literature from the target's perspective. In particular, we focus on how diversity ideologies—beliefs or organizational practices with regards to how to approach diversity—affect racial minorities' and women's self-perceptions and experiences at work. This review suggests that a diversity aware ideology (i.e., multiculturalism) is more beneficial than a diversity blind ideology (i.e., colorblindness) for racial-ethnic minorities (e.g., better performance outcomes; more psychological engagement, inclusion, and workplace satisfaction; more positive leadership self-perceptions; and reduced perceptions of bias and turnover intentions). In contrast, for women, gender-blindness is associated with more positive outcomes than gender awareness (e.g., enhanced self-confidence, pro-active behaviors and leadership emergence). Importantly, multiculturalism and gender-blindness can both produce negative side effects for racial minorities and women, respectively, which highlights the importance of developing approaches to address the shortcomings of these conventional ideologies. We discuss the implications and offer recommendations for future research.

Over the last decades, racial and gender diversity in organizations has strongly increased. Enhanced diversity has the potential to give rise to positive outcomes in organizations such as creativity and effectiveness in workgroups (Homan et al., 2007; Page, 2007; Barta et al., 2012). On the flipside, diversity also has the potential to increase negative organizational outcomes such as conflict and miscommunication (Pelled et al., 1999; Van Knippenberg et al., 2004). As a consequence of diversity's potential to be both beneficial and harmful, many organizations have sought to understand how to leverage the upsides and manage the downsides (Galinsky et al., 2015). A key challenge and opportunity in this process is understanding the psychology of traditionally underrepresented groups such as women and racial-ethnic minorities in response to diversity initiatives.

In attempts to effectively manage diversity, many companies utilize structural and institutional initiatives, such as affirmative action, but also diversity training, and official diversity policies (Konrad and Linnehan, 1995; Kelly and Dobbin, 1998; Ely and Thomas, 2001; Jackson et al., 2003; Kalev et al., 2006; Leslie et al., 2014; Hideg and Ferris, 2016). Such initiatives can increase the representation of women and racial-ethnic minority employees in the short-term; however, over the long-term, their effectiveness has been shown to be limited. Indeed, racial minorities and women remain underrepresented in the upper echelons of organizational power (Catalyst, 2016; Fortune, 2017). Further, although this research examines representational outcomes, these interventions are often targeted at those in power (managers; e.g., affirmative action, policies) or majority group members (Whites, men; e.g., bias). Much less work has focused on the psychological experience of underrepresented groups in reaction to these policies. Indeed, past work has shown that though certain initiatives (such as affirmative action) can have a positive effect of representation (Crosby et al., 2006; Kalev et al., 2006), the psychological experience on those groups can often be negative, whereby they become targets of prejudice (Leslie et al., 2014; Hideg and Ferris, 2016) and question their efficacy at work (Heilman et al., 1987). Thus, the experience of underrepresented groups may be very different from their representational outcomes.

In addition to diversity initiatives targeting organizational structures, organizations can also utilize complementary approaches “to shape the cultural context of the workplace” (Apfelbaum et al., 2016, p. 547). Given the potential downsides of structural initiatives and significance of examining the experiences of underrepresented groups in reaction to diversity initiatives, understanding these complementary approaches and their impact on racial minorities' and women's attitudes, cognitions and behavior remains important (Joshi, 2014; Apfelbaum et al., 2016). One of the most prominent among these are *diversity ideologies* (Wolsko et al., 2000; Apfelbaum et al., 2016). Diversity ideologies can refer to organizational practices that are often explicitly summarized in a diversity mission statement and communicate the organizational approach to and norms around diversity. In addition, diversity ideologies can also refer to employees' own beliefs around how to approach group differences in diverse settings (Martin and Phillips, 2017). Thus, ideologies can be contextual or individual (or both).

In this review, we examine diversity ideologies, which have been shown to promote diversity and inclusion in organizations (Wolsko et al., 2000; Rattan and Ambady, 2013; Sasaki and Vorauer, 2013; Plaut et al., 2018), and their effects on racial minorities' and women's experiences in organizations. In doing so, we focus our analysis on two levels: ideologies as contextual or organizational level variables (i.e., imposed by the organization or those in power) and as individual level variables (i.e., beliefs held by individuals). We review these two levels, as they are mutually reinforcing, where organizational beliefs can be adopted by individual members (Bourguignon, 2017; Martin and Phillips, 2017), and individual beliefs can shape organizational cultures (Schein, 1992)^1^. Our review of research within organizational settings elucidates why minorities or women respond differently to different ideologies, and have unique outcomes in similar ideological contexts. Moreover, although our main goal is illuminating workplace behavior and outcomes, we also discuss research in adjacent areas (e.g., stereotyping, prejudice, interaction) that offers complementary insights relevant for organizations.

The current contribution reviews and synthesizes existing literature in a systematic way to highlight the role of diversity ideologies on traditionally underrepresented groups' in particular, racial minorities and women) self-perceptions, experiences and behaviors in diverse work settings. Doing so makes at least two broad contributions. First, in previous work, diversity ideologies have gotten ample attention in many areas of research, ranging from educational to government policy. Adding a comprehensive review on their role in the *organizational context* is valuable, as it not only theoretically clarifies the types of organizational ideologies that benefit the very groups they aim to help, but also gives practical advice for organizations looking to understand the messages they use to reach that goal. That is, identifying conceptual confounds and ambiguities around ideological messages is important to understand how to effectively implement them in organizations. For organizations looking to increase and improve the dynamics around diversity, this can then help increase the status and resources amongst underrepresented groups (an important precursor to societal equality). Second, by combining ideologies literature focusing on both racial minorities' and women's perspective, this review integrates two lines on inquiry that have primarily developed in isolation. As such, this work allows us to uncover similarities and differences of racial minorities' and women's responses to different ideologies. Below, we first define the dominant ideologies in the literature. Our initial discussion of (variations in) different ideologies focuses primarily on the context of race-ethnicity as there is more information available in this domain, and thus, offers the richest information. Here, we also pay some attention to the conceptualization of gender ideologies, which can be seen as a continuation of the race-ethnicity literature. We zoom into ideologies' impact on racial minorities, followed by their impact on women' experiences. In our integrative discussion we identify patterns and shortcomings in the literature and propose key future directions.

## Diversity Ideologies: Blindness vs. Awareness

As a consequence of continuously diversifying society, academics have sought to find ways to better understand intergroup relations. These attempts have traditionally focused on stereotyping, discrimination as well as representational concerns around traditionally underrepresented groups (Fiske et al., 2002; Crosby et al., 2006; Kalev et al., 2006). An alternative to these traditional foci is to illuminate the role of organizational practices or individual beliefs around how to approach diversity in the quality of intergroup relations. These practices or beliefs, diversity ideologies, are highly consequential and offer a complementary way of uncovering the dynamics around and outcomes of intergroup contact (Wolsko et al., 2000; Rattan and Ambady, 2013).

Most research on diversity ideologies has been done in the context of race. Existing work identifies two broad types of diversity ideologies, which differ in the extent to which they recognize or ignore differences between demographic groups. Though they differ in their approach, the two dominant ideologies share the same ultimate goal: contributing to an environment in which diverse groups of people can harmoniously live and work together (e.g., Wolsko et al., 2000). One ideology aims to do that by ignoring and de-emphasizing differences between groups, while the other takes the opposite approach, by being open to and recognizing differences.

One type of ideology, the so-called *colorblindness* (i.e., blindness) approach, focuses on de-emphasizing differences between social groups (Wolsko et al., 2000; Apfelbaum et al., 2012). The underlying assumption of this ideology is that categorizing individuals by their social group leads to prejudice and conflict. Thus, ignoring social categories should reduce these negative consequences. The colorblind ideology is not without its critics. Opponents of colorblindness suggest that suppressing social categories is not possible, as humans have a natural tendency to categorize their environment to be able to process the large amount of information (Rosch and Lloyd, 1978). Moreover, research shows that demographic group information, like race and sex, is detected in the brain within milliseconds (Ito and Urland, 2003). Opponents propose that colorblindness is not only impossible but also undesirable because it ignores the unique cultural identities and traditions of racial minorities and assimilates them into a dominant power structure (Fryberg and Stephens, 2010). Further, diversity has the potential to offer positive contributions to companies and the society as a whole (Van Knippenberg et al., 2004). As such, opponents of colorblindness argue that differences between demographic groups should not be ignored but recognized and celebrated.

The idea that diversity should be emphasized rather than ignored is central to the second prominent ideology in the literature, *multiculturalism* (i.e., awareness). In this view, differences between social groups should not only be recognized and emphasized, but also valued and celebrated. Proponents of multiculturalism argue that categorization does not have to be harmful (Park and Judd, 2005; Costa-Lopes et al., 2014). When differences between demographic groups are perceived in a positive manner (e.g., as sources to learn from), they do not evoke prejudice. Moreover, demographic group differences can be meaningful and important to the members of these groups; ignoring or undervaluing of which would do these groups a disservice. However, similarly to colorblindness, this view also has its critics, who argue emphasizing differences between groups can exacerbate stereotypes, create divisions between groups, delegitimize racial inequity claims, and promote racial segregation (Verkuyten, 2005; Hahn et al., 2010, 2015; Gündemir and Galinsky, 2018).

Like diversity ideologies focusing on race-ethnicity, gender ideologies also differ in the extent to which they recognize vs. overlook intergroup differences. On the one hand, there is *gender-blindness*. Analogous to colorblindness, this view proposes that the differences between men and women are neither meaningful nor consequential and thus they should be ignored and men and women should be treated as individuals (Koenig and Richeson, 2010; Martin and Phillips, 2017). On the other hand, there is *gender awareness*. Analogous to multiculturalism, gender awareness proposes that differences between men and women should be recognized and celebrated.

## Conceptualizing Different Forms of Diversity Ideologies

While many agree that the dichotomy between de-emphasizing vs. acknowledging and celebrating social group differences is common across studies on diversity ideologies (Hahn et al., 2015), it is noteworthy that both ideologies are complex and can take different forms. Recent work has elaborated on the importance of *how* these ideologies are conceptualized and measured. With regards to the blindness ideology, scholars have depicted this approach in multiple ways, depending on different intentions toward the outgroup (e.g., assimilation vs. inclusion; Hahn et al., 2015) and differences in the focus of attention (e.g., sameness vs. de-emphasis of subgroup differences in favor of individual uniqueness; Rosenthal and Levy, 2010).

For example, regarding intentions toward the outgroup, Hahn et al. (2015) note that while conceptions of blindness converge in their de-emphasis of difference, an assimilationist approach entails that such “sameness” should be defined by the superordinate group's norms (e.g., “organizations should encourage racial minorities to adapt to mainstream ways”; Plaut et al., 2009), whereas an inclusion-focused colorblind approach de-emphasizes difference make minority groups feel included (e.g., “you can find commonalities with anyone no matter their background”; Hahn et al., 2015).

Further, with regards to which differences are the focus of attention, some blindness ideologies focus on recognizing sameness whereas others focus on individual differences. That is, colorblindness has been portrayed as a *value-in-homogeneity* approach, in which differences between groups are suppressed in favor of an overarching group membership (Plaut et al., 2011; Holoien and Shelton, 2012; Todd and Galinsky, 2012; Gaertner and Dovidio, 2014). However, colorblindness has also been depicted as a *value-in-individual differences* approach, focusing on ignoring any type of group membership (e.g., a subgroup or an overarching one) in favor of individual qualities that make people unique (Wilder, 1984; Ryan et al., 2007; Verkuyten, 2010; Peery, 2011).

Regarding these different conceptualizations of colorblindness, scholars have debated whether these different conceptualizations represent subtypes of the colorblind ideology or separate ideological approaches (Rosenthal and Levy, 2010). Some work has labeled assimilation as a separate ideology from colorblindness, as unlike the benevolent nature of colorblindness, assimilation's sameness-focus perpetuates the dominant group's norms (e.g., Hahn et al., 2015). Others have suggested that value-in-homogeneity and value-in-individual differences are subtypes of colorblindness, as both variants are characterized by a lack of recognition of subgroup differences, (e.g., Dovidio et al., 2010; Gündemir et al., 2017a). These latter scholars also recognized that while the psychological consequences of the salient subtypes may differ, many common manipulations and measures of colorblindness integrate elements from both (Wolsko et al., 2000; Ryan et al., 2007), making it unclear which element is causing or creating the effects. Finally, most recently, it has been suggested that colorblindness can also be interpreted as *value-in-equality*, which focuses on a meritocratic perspective of equivalent and fair treatment of different social groups (Apfelbaum et al., 2016).

Similar to the multifaceted nature of colorblindness, conceptualizations of the awareness ideology have also differentiated between the intentionality and focus of an ideology that highlights group differences. For example, some work differentiates between the positive version of *multiculturalism* (recognition and preservation of category distinctions to build a strong, diverse community) and *negative* version of segregation (the separation of groups, such that they occupy different spheres). Further, multiculturalism can be interpreted as the celebration of cultural differences (Wolsko et al., 2000; Government of Canada, 2018), or the inclusion of different cultural backgrounds into an environment (Markus et al., 2000; Apfelbaum et al., 2016), as well as respect for cultural differences and identities (Markus et al., 2000; Purdie-Vaughns and Walton, 2011). In these latter conceptualizations, it is unclear whether the benefits of multiculturalism are due to the celebration, inclusion, or respect of differences, and future research is needed to better disentangle these effects.

Similar to the conceptualization challenges in race-ethnicity research, the yet limited amount of work on gender ideologies is also confronted with conceptualization issues. Analogous to colorblindness, measures of gender-blindness often include both a *value-in-individual differences*, focusing on individual differences between men and women, and a *value-in-homogeneity*, focusing on emphasizing what is common among men and women (e.g., Koenig and Richeson, 2010; Martin and Phillips, 2017). Unlike the clearer conceptual distinctions made in research on race ideologies, empirical research in this domain has rarely distinguished between these components. While these conceptualizations represent hierarchy attenuating ideologies, some conceptualizations are hierarchy enhancing, such as a gender-blind approach which focuses on women adapting to men, which is consistent with assimilation (Hahn et al., 2015). Similarly, some work argues that some forms of gender-awareness are akin to segregation, which aims to keep men and women in separate domains (e.g., jobs, schools; Hahn et al., 2015).

In sum, although some work distinguishes between different forms of colorblindness and multiculturalism (e.g., Verkuyten, 2010; Levin et al., 2012; Hahn et al., 2015), much of the existing research operationalizes colorblindness and multiculturalism in ways that integrates elements from each variant (Wolsko et al., 2000; Gutiérrez and Unzueta, 2010; Morrison and Chung, 2011). Similarly, conceptualizations of gender ideologies often involve elements from different variants. As such, when evaluating the effectiveness of colorblindness and multiculturalism or gender-blindness and gender awareness, it can be hard to determine which elements are responsible for the observed effects. It is important to note that conceptualization of the blindness ideology is typically more variable than that of the awareness ideology, hence, we pay more attention to specifying the type of blindness in our discussion of empirical findings below.

Below, when the reviewed work specifies the exact conceptualization of color/gender-blindness, we make note of which conceptualization was used; otherwise, when left unspecified or if multiple elements occur simultaneously, we use the overarching term “colorblindness” or “gender-blindness” for race and gender ideologies, respectively.

## How Do Racial Minorities Respond to Diversity Ideologies?

To understand minorities' responses to diversity ideologies, Dovidio and colleagues offer a functional perspective. In this perspective, the responses of minorities to different ideologies are thought to be an outcome of the extent to which each ideology addresses their group based needs (Dovidio et al., 2007, 2010). The salient ideology gives the members of different groups signals about how comfortable they can feel within, and how much they can trust, a given environment (Purdie-Vaughns et al., 2008). By ignoring—and therefore seemingly not valuing group differences—a blindness ideology overlooks group-based challenges minority groups may experience and allows the majority group to maintain their dominant position. An awareness ideology, however, acknowledges the minority group's need for group-based recognition and appreciation and can help change the status quo (by, for example, making conversations about group based disparities less a taboo, cf., Schofield, 2001, Saguy et al., 2009), enhancing the position of the minority group. As such, an awareness ideology (i.e., multiculturalism), could be more functional for the minority group and this may be especially true for those who strongly identify with their group (Verkuyten, 2005, 2009). Below, we review empirical research concerning the link between diversity ideologies and responses of racial minorities.

### Empirical Work on Racial Minorities' Responses to Diversity Ideologies

Research on the impact of diversity ideologies on racial minorities focuses broadly on three areas: (1) minority group members' preference for different ideologies, (2) the effects of dominant group members' ideology on minorities' responses and experiences, and (3) the role of ideologies at the organizational level on minorities' perceptions and behavior. We discuss relevant findings next.

In line with the previous arguments, empirical work demonstrates that the members of minority groups have a strong preference for multiculturalism (Markus et al., 2000; Arends-Tóth and Van De Vijver, 2003; Wolsko et al., 2006; Ryan et al., 2007, 2010). Consistent with the functional perspective, a preference for multiculturalism likely stems from minority groups' desire for their group-based needs to be recognized. Supporting this idea, a preference for multiculturalism is not solely unique to racial minorities, it applies to *any* group that holds minority, subordinate status within a given environment. For example, White students at predominantly black colleges, where they are the representational minority and hold a lower status position, endorse diversity aware policies in these institutions. That is, they prefer that their group be recognized and their needs be addressed. However, these same students endorse diversity blind, assimilationist policies at the national level, where their group is the representational majority and holds the higher power and status position, as their group is already recognized and needs already addressed (Hehman et al., 2012). This finding suggests that as the functionality of an ideology shifts, so do groups' preferences.

Additionally, research has shown that, by creating a specific climate, the diversity ideology endorsed by the majority group can have important consequences for minorities' perceptions and experiences. When the majority group endorses multiculturalism (rather than colorblindness), racial minorities tend to perceive less bias, and experience more engagement and inclusion. For example, Plaut et al. (2009) studied minorities' psychological engagement at work (i.e., the extent to which employees value work success and organizational membership) in response to their majority group co-workers' ideology. This work showed that to the extent that the majority group employees endorse multiculturalism in a unit, minorities report higher psychological engagement. The majority group's endorsement of colorblindness (measured as assimilation), is associated with reduced psychological engagement among minorities. This positive effect of multiculturalism on minorities' engagement is explained by perceptions of bias. That is, minorities experience less racial bias when the climate is characterized by multiculturalism, which in turn boosts their psychological engagement.

This result is consistent with experimental research, which showed that racial minorities experience more engagement on a cognitive task when interacting with majority group counterparts who are primed with multiculturalism rather than colorblindness (Holoien and Shelton, 2012). This greater engagement exhibited by racial minorities is explained by perceptions of lesser bias from their majority group partners (Holoien and Shelton, 2012). Similarly, in workgroups, minority employees feel more accepted to the extent that leaders endorse multiculturalism (vs. colorblindness), which results in more effective workgroup functioning (Meeussen et al., 2014). Finally, Vorauer et al. (2009) found that compared to a colorblind ideology, the majority group (in this case White Canadians) primed with a multicultural ideology show more engagement in minorities (in this case Aboriginal Canadians), which leads minorities to have fewer evaluative concerns and experience less anxiety. With regards to racial minorities' performance in organizations, research suggests that compared to colorblindness, an awareness (i.e., multicultural) ideology can improve the performance of racial minorities on cognitive tasks (Wilton et al., 2015; Apfelbaum et al., 2016).

Not only does multiculturalism seem to benefit racial minorities when Whites adopt this ideology, but similarly, when the (organizational) context is characterized by multiculturalism (through, for instance, diversity mission statements), minorities also experience positive outcomes. For example, minorities' perceptions of organizational multiculturalism can boost their workplace satisfaction, by enhancing their sense of inclusion within an organization (Jansen et al., 2016). Further, multiculturalism can reduce minorities' turnover intentions, especially when they strongly identify with their cultural-ethnic group (Phouthonephackdy, 2016). Additionally, research in Western Europe showed that diversity aware environments can enhance religious minorities' positive perceptions of education and work (Van Laar et al., 2013). Moreover, Purdie-Vaughns and Eibach (2008) found that when African American professionals are attuned to minority representation, workplaces that espouse a colorblind, value-in-homogeneity message leads them to perceive threatening identity contingencies and to distrust of their organizational environment.

One study has extended these findings to the context of leadership. Because minorities remain underrepresented in higher leadership positions, it is important for organizations to find ways to stimulate minority leadership (e.g., Ospina and Foldy, 2009; Gündemir et al., 2014). Some scholars wondered whether organizational diversity ideologies can stimulate minority leadership by boosting their leadership self-perceptions (Gündemir et al., 2017a). This work showed that, by communicating an open diversity climate, multiculturalism can indeed help minorities to cultivate more positive leadership self-perceptions. When organizational diversity mission statements communicate multiculturalism, minorities report increased leadership self-efficacy (i.e., the extent to which they think they are able to fulfill leadership tasks successfully) and stronger leadership aspirations (i.e., intentions to apply for leadership roles) than when the value-in-homogeneity variant of colorblindness is salient. Interestingly, this work did not find a difference between multiculturalism and the value-in-individual differences variant of colorblindness. The authors suggested that the value-in-individual differences variant of colorblindness' acknowledgment of differences, albeit at the inter-individual level, may -to some extent- address minorities' need for recognition of differences and thus be more “functional” for them than the value-in-homogeneity variant.

Together, empirical work suggests that minorities respond more positively to (organizational) contexts characterized by multiculturalism (rather than those characterized by blindness) and these contexts appear to improve task engagement amongst minority groups.

### Additional Considerations Around Minority Responses to Diversity Ideologies

It should be noted that there are several important contextual factors with respect to the above-depicted effects. In most of this research, racial minorities represent a small number in organizations and prefer multiculturalism over colorblindness. However, these effects often depend on representation, the ways in which racial minorities perceive the messages being espoused, and the types of differences being highlighted. For example, in the few contexts where they represent the majority group, past work has found African Americans prefer an assimilationist, blindness approach, as their identities are already valued and embraced (Hehman et al., 2012). Further, while much research suggests racial minorities prefer and perform better with multiculturalism, recent work suggests that when minority groups are strongly underrepresented (e.g., making up about 5% of the company) they may wish to merge with the rest (Apfelbaum et al., 2016). In those circumstances multiculturalism may be less effective for performance.

Moreover, multiculturalism can produce some unintended side effects. For example, Zou and Cheryan (2015) note that when multiculturalism is highlighted, racial minorities may feel a “minority spotlight effect,” leading them to experience a heightened sense of self-awareness, negative emotion, and discomfort (Crosby et al., 2014). Further, multiculturalism can lead racial minorities in the U.S. to feel excluded from the overarching national identity (e.g., the American identity), lowering their motivation and self-esteem (Zou and Cheryan, 2015). Consistent with this, Verkuyten (2005, 2009) showed that multiculturalism was only related to heightened self-esteem among those for whom their racial-ethnic identity is highly salient and not among those for whom their racial-ethnic identity is less salient. Multiculturalism can also lead to an emphasis of certain, sometimes problematic, differences. For example, multiculturalism has been shown to increase race essentialism (Wilton et al., 2018, but see Martin, 2018) and lead to (positive) stereotyping of the racial minority group (Gutiérrez and Unzueta, 2010), which can lead to negative reactions and perceived prejudice amongst racial minorities (Czopp, 2008). Moreover, although interpreted positively by racial minorities, majority groups perceive the pro-diversity attitudes communicated through multiculturalism as exclusionary (Plaut et al., 2011), subsequently limiting their support for organizational diversity efforts, which can have negative spillover effects on racial minorities' experiences.

One additional side effect of multiculturalism is that it can create a false fairness context. Gündemir and Galinsky (2018) demonstrated that minority group observers perceive organizational diversity mission statements characterized by multiculturalism as a cue for fair treatment of minorities. This, in turn, is associated with disregarding of information about potential racial discrimination and delegitimization of racial discrimination claims (Kaiser et al., 2013; see also Dover et al., 2016).

In sum, although the discussed literature until now shows the promise of multiculturalism vs. colorblindness from the perspective of racial-ethnic minorities, it also indicates some contingency factors for its effectiveness and even some potential downsides. Recently, researchers have started exploring the role of diversity ideologies for another key demographic group: women. Below, we review this work and contrast those findings with the findings around race-ethnicity.

## How Do Women Respond to Diversity Ideologies?

In contrast to much research on the benefits of an awareness ideology, multiculturalism, for race, research shows that gender-blind ideology may be beneficial for women. One possible reason for this discrepancy is that the differences made salient for race through multiculturalism tend to be those focused on cultural identities and experiences of racial minorities, which are often ignored in a power structure that is frequently dominated by the majority group. For gender, the types of differences made salient through an awareness ideology are those that focus on stereotypical gender roles, including personality, skill, and preference differences (i.e., men as agentic; women as communal; Martin and Phillips, 2017, 2019; Martin, 2018). Since agentic qualities overlap with leadership qualities, gender-blindness may be more appropriate in the work domain, because reducing sexism involves seeing women as capable and competent with regards to their leadership abilities and potential (Martin et al., 2016).

How do the gender aware vs. gender-blind ideology impact workplace perceptions and outcomes of women? Martin and colleagues suggest that the gender-blind ideology is more instrumental for women at work than the gender aware ideology as the latter can emphasize traditional differences in social roles associated with men and women (also see Eagly and Karau, 2002; Martin et al., 2016). Social role theory suggests that, as a consequence of traditional role distribution between men and women, different group based stereotypes of men and women have emerged (Eagly, 1997). Women are typically associated with communality (characteristics such as warmth and consideration) and men with agency (characteristics such as self-confidence and dominance; Eagly and Karau, 2002). This perceived dichotomy can stand in the way of women's career development because higher status and leadership roles are more strongly associated with agency than communality.

Though this area of research is nascent, theory suggests that gender-blindness can have a positive impact on women at work. Because gender awareness can heighten the salience of the communality of women vs. agency of men, a gender-blind ideology may be more effective for women (Martin et al., 2016; Martin and Phillips, 2017).

### Empirical Work on Women's Responses to Diversity Ideologies

Thus far, empirical research on the impact of gender ideologies on women is limited and focuses broadly on (1) women's preference for ideologies and the role of ideologies held by individuals on women's experiences, (2) the effects of ideologies held by the dominant group members (i.e., men), and (3) ideologies at the organizational level affecting women's experiences. We discuss relevant findings next.

Early research has shown that women (as well as men) perceive gender-blind ideology as more appropriate in the work domain (Koenig and Richeson, 2010). According to this work, gender-blindness is perceived as a way to reduce sexism. Outside of the workplace, where men and women often exist in dyadic, interdependent, and familial relationships, an awareness ideology is preferred (Koenig and Richeson, 2010). However, in the workplace, where women face sexism-related challenges, blindness is seen as more fitting in the workplace. Further, some work shows that endorsement of gender-blindness is negatively related to biological essentialism (Martin, 2018), while others reported non-significant effects (*r* = −0.09, *p* < 0.10; Hahn et al., 2015). However, the reported negative effects are specific to hierarchy-attenuating forms of gender-blindness (*value-in-individual differences*, and *value in homogeneity*), as hierarchy-maintaining (assimilationist) forms of gender-blindness are associated with endorsement of essentialist beliefs (Hahn et al., 2015). Thus, it is clear that the conceptualization of gender-blind ideologies is an important factor in explaining these effects.

The relationship between gender ideologies and essentialist beliefs is problematic, as gender-essentialism is related to more stereotyping, sexism, and backlash (e.g., Martin and Parker, 1995; Bastian and Haslam, 2006); thus, it appears that gender-blindness may have the potential to lessen sexism women experience. Indeed, Koenig and Richeson (2010) found that gender-blindness is negatively associated with sexism, both in individuals' desire to respond without, and benevolent sexism (a form of sexism which denies women agency, by seeing them as reliant on men; Glick and Fiske, 1996). Importantly, the relationship between gender-blindness and benevolent sexism is not limited to men. Women's own endorsement of benevolent sexism leads to a host of problems, where exposure to, and endorsement of, benevolent sexism leads to lower achievement efficacy (Barreto et al., 2010), performance on male-typed tasks (Vescio et al., 2005), and preference for more dependent, and less autonomous, help (Shnabel et al., 2016). Overall, these findings suggest that gender-blindness, particularly when conceptualized as value-in-individual differences or value in homogeneity instead of assimilation, has the potential to create contexts where women experience less sexism.

Some studies examined the role of men's adoption of gender ideologies on women's responses. Martin and colleagues found that when men were primed with gender-blindness, they reduced their dominance in interactions, leading women to contribute more to the conversation (through increased talking time; Martin et al., 2016). Recent research suggests that men who endorse or are exposed to gender-blind messages are less likely to endorse gender-STEM stereotyping, with downstream consequences for evaluation of female scientists, both of which have previously been shown to limit women's opportunities in STEM (Martin and Phillips, 2019). Also, men who were primed with gender-blind ideologies were also more likely to support affirmative action policies, which help women advance in environments where they are underrepresented (Martin, 2018).

Further, when the (organizational) context is characterized by gender-blindness, it appears to be beneficial for women as well. Research showed that women in a gender-blind setting report higher levels of self-confidence, especially in male dominated environments (Martin and Phillips, 2017). Moreover, this increased self-confidence leads them to act in more pro-active ways (e.g., taking more risks), which are actions and behaviors needed to be successful in many work environments and positions of power.

Taken together, although the current state of knowledge on gender ideologies is limited, existing work suggests that gender-blindness may be beneficial for women's advancement at work.

### Additional Considerations Around Women's Responses to Diversity Ideologies

Though nascent research has found positive effects of gender-blindness on women's workplace outcomes, like multiculturalism on racial minorities' outcomes, these effects seem to be contextual as well. For example, Martin and Phillips (2017) found that the benefits of gender-blindness are limited to those where men represent the majority and women are underrepresented. In fact, in communal environments (or those made up of majority women) gender-awareness seems to be more effective. Apfelbaum et al. showed similar effects; when women represent a substantial percentage in an organization (40%) they prefer a *value-in-difference* approach. Martin et al. (2018) uncovered that in fact, it is only women who have strong career values (i.e., those who prioritize career related goals) who prefer gender-blindness. Conversely women who have stronger family values (i.e., those who prioritize family related goals) actually prefer gender-awareness.

Further, gender-blindness (much like multiculturalism) can create its own negative side effects. For instance, policies such as “meritocracy,” which many companies utilize as a form of the blindness ideology (Apfelbaum et al., 2016), that ignore factors that shape and bias women's performance at work (i.e., being “blind” to these issues) exacerbate prejudice toward women in occupational domains. In this respect, Castilla and Benard (2010) show that the presence of meritocratic (i.e., gender-blind) policies prompt both male and female decision makers to offer higher levels of bonus to men than to equally qualified women. The authors speculate that these gender-blind policies can, for instance, enhance moral credentials of decision makers, which in turn evoke biased decision-making. The same study also demonstrates that when the context communicates awareness for biases women at work face, decision makers can engage in behaviors that attempt at making up for injustice. Thus, by reducing awareness of group-based challenges women face, gender-blindness may be detrimental for their workplace experiences and outcomes. Finally, gender-blindness can exacerbate backlash for women who display more feminine behavior (Malicke, 2013). Thus, there is potential for gender-blindness to prohibit women from behaving in stereotypically feminine ways, which may mute their authenticity. Although these current insights primarily highlight how women (vs. men) are perceived as targets rather than highlighting the target's own perspective, these findings are informative for understanding the potential downsides of the gender-blind ideology and form a stepping stone for future work extending these to the target's perspective.

## Suggestions From Past Research to Address the Shortcomings of Diversity Ideologies

The discussion above suggests that multiculturalism may be beneficial for racial minorities and gender-blindness for women. At the same time, it demonstrates that both multiculturalism and gender-blindness can have unintended, negative consequences. This has led many scholars to attempt to develop more nuanced ideological approaches to diversity, primarily in the context of racial diversity.

Some scholars suggested a focus on “identity safety,” rather than multiculturalism. The identity safety approach acknowledges that diversity can be a source of value and that social groups *can* experience social contexts in similar ways, but that various barriers may prevent them from doing so (Purdie-Vaughns and Walton, 2011). Others proposed that, to reduce the majority group's sense of exclusion, multiculturalism message should explicitly include the majority group in it, the so-called *all-inclusive multiculturalism* approach (Stevens et al., 2008; Plaut et al., 2011). This could lower their resistance among the majority, creating more inclusive environments where minorities have more opportunities and more positive work place experiences.

Another strategy introduced in recent research has focused on ways to reduce the negative effects of multiculturalism while retaining its positive effects. This work demonstrated that explicitly incorporating an equal opportunity, value-in-merit message to multiculturalism can help circumvent some of multiculturalism's negative effects (Gündemir et al., 2017b). This synergistic approach termed *multicultural meritocracy* emphasizes organizations' commitment to a highly accomplished, qualified and diverse workforce. Multicultural meritocracy reduces negative effects of multiculturalism such as stereotype activation of minorities and sense of exclusion by the majority, while retaining its positive effects such as psychological engagement of minorities (Gündemir et al., 2017b).

Although research on such ideal strategies is missing in the context of gender ideologies, we speculate that this last approach may also help address some of the shortcomings identified in gender ideologies research. The synergistic approach of gender aware meritocracy may tackle some of the specific limitations of the gender-blind ideology. For example, Martin (2018) found that compared to a generalized “awareness” message, an “experience-awareness,” which included examples of experiences of women, increased men's recognition of discrimination and increased their support for affirmative action policies. By focusing on the unique experiences and obstacles women face, rather than essential, gender-role differences, men's attention was directed toward the differences often highlighted for race through multiculturalism, and away from gender-role stereotypes that limit women's opportunities. Thus, adding gender awareness (i.e., awareness of experience) to the gender-blind (i.e., blindness to essentialist differences), meritocratic message can make decision makers aware of the potential for gender-based prejudice, which can reduce biased decision making in reward distribution (see Castilla and Benard, 2010). Moreover, since gender aware meritocracy provides a more inclusive message than the gender-blind ideology, in which gender based differences are not only recognized but also explicitly valued, engaging in typically feminine behaviors may be more accepted (see Malicke, 2013). Thus, such a gender aware meritocracy message may be more effective than gender-blindness as it is less likely to ignore gender bias and to prohibit women from behaving in feminine ways.

In sum, although multiculturalism and gender-blindness appear to be promising for racial minorities and women, respectively, neither ideology is a panacea as both can create negative side effects. One alternative approach, multicultural (or gender aware) meritocracy, has been shown to be beneficial for racial minorities and has the potential to benefit women. More research is needed to understand effective strategies for successful implementation of diversity ideologies.

## General Discussion

We have presented a review of the diversity ideologies literature from the target's perspective. In particular, our discussion of the literature focused on the target's perspective, highlighting how the diversity ideology affects racial minorities' and women's self-perceptions and behaviors in work settings. The literature suggests that a diversity aware, multiculturalism ideology, which recognizes and celebrates social group differences, is associated with more positive outcomes than a diversity blind, colorblindness ideology for racial-ethnic minorities, such as better performance outcomes, increased psychological engagement, inclusion, and workplace satisfaction, more positive leadership self-perceptions and reduced perceptions of bias and turnover intentions. For women, gender-blindness ideology is associated with more positive workplace outcomes than a gender aware ideology, such as enhanced self-confidence, pro-active behaviors and leadership emergence.

Taken together, the patterns around race-ethnicity vs. gender present a conundrum for researchers and practitioners. In general, diversity-awareness appears to be effective for some target groups of diversity initiatives such as racial minorities, whereas diversity blindness is more effective for other target groups such as women. Where does this discrepancy come from? Existing theory and empirical work suggest that racial minorities have a group-based need to be acknowledged and valued for their differences (Dovidio et al., 2010). Hence they respond more positively to the awareness ideology. For women, however, an increased awareness of gender differences may activate stereotypes, which may stand in the way of their career development (e.g., Martin and Phillips, 2017). As such, for them a blindness ideology may be more instrumental and thus evoke more positive responses. Especially given the finding that most (about two thirds of) companies utilize a diversity aware approach (Apfelbaum et al., 2016), our review suggests that while these approaches are potentially beneficial for racial-minorities' career development, they are unlikely to be effective for women's career development. Consequently, organizational leadership needs to clearly specify the target group(s) of their diversity approach and tailor their approach to address different groups' position and needs.

While it is unlikely that one, holistic approach to diversity is the solution to these problems, the underlying reasons that diversity ideologies seem to have different effects on racial minorities and women are the types of differences being embraced and downplayed through race/gender awareness (Martin, 2018). As suggested above, perhaps a more nuanced approach, which specifies the types of differences to be “aware of” or “blind to” and how to implement these solutions effectively could be more effective in providing benefits to both racial minorities, women, and even other social groups. In line with *identity safety* (Purdie-Vaughns and Walton, 2011)—highlighting the similarities between social groups, while acknowledging their different experiences in social settings—there may be potential to leverage the potential of both of these ideologies. Consistent with this, a multicultural meritocracy approach, which simultaneously emphasizes value in diversity and value in merit, may offer a promising new way for both race-ethnicity and gender diversity (Gündemir et al., 2017b).

### Implications for Racial Minorities

Given the growing racial diversity (e.g., Colby and Ortman, 2015; Eurostat, 2018), it is important to understand the effects of how to navigate and leverage this diversity. Our review suggests that multiculturalism can be an effective strategy in making racial minorities feel included, empowered, and engaged. In contrast to organizations that are inclined to favor one ideology over the other (often awareness; Apfelbaum et al., 2016), individuals are more likely to simultaneously endorse both aware and blind ideologies (often equally; MTV Bias Survey, 2014; Hahn et al., 2015). These dynamics make it important to ensure that research extends beyond the lab to the field. In doing so illuminating the interaction between organizational and individual diversity ideologies is of key importance. Further, it is important to understand when, where, and why multiculturalism is beneficial to racial minorities as some work suggests that these results are specific to environments where racial minorities are underrepresented, identify with their race to some extent, and do not feel a heightened self-consciousness based on such “awareness.”

While this review focused on the effects for racial minorities, it is equally important to understand how these approaches affect dominant group members own sense of efficacy, inclusion, and performance. Indeed, some research has suggested that multiculturalism makes Whites feel excluded, which can in fact undermine their efficacy and performance. Thus, as organizations attempt to implement these strategies, an inclusive multiculturalism strategy becomes increasingly important to best leverage diversity of all organizational members, and not just the minority group. Increasing a sense of inclusion for all groups can also have direct benefits from the target's perspective as research suggests that this would encourage the majority or the dominant group to endorse pro-minority initiatives in organizations (Plaut et al., 2011).

### Implications for Women

In contrast to the benefits for racial minorities, this review also indicated that gender-blindness seemed to be a more effective approach for women in organizational domains. Given the dominant approach to organizational diversity is an awareness approach (Apfelbaum et al., 2016), it is important that the implications of this approach for women is also considered. Indeed, scholars have assumed that awareness ideologies are beneficial for all social groups (Plaut, 2010; Galinsky et al., 2015). However, it seems like this may not always be true, making it increasingly important to understand the unintended consequences of these ideologies for women, as well as other social groups. Additionally, many in the public and practitioner sphere embrace awareness ideologies, advocating for women to “own it” and embrace their femininity and feminine qualities at work to be successful at work (Annis and Merron, 2014; Krawcheck, 2017). There are far fewer books in popular culture advocating for a gender-blind approach; thus, it becomes important to heed caution in promoting these strategies, without knowing their implications for women.

Further, this review found that gender-blindness seems to be more effective than awareness in male dominated domains, positions of power, or for women who value their career quite strongly. Thus, it is important to understand the limits to these effects, as downplaying gender differences may also have potential to blind people to women's unique experiences in organizations or prohibit women from engaging in feminine behaviors (perhaps making women feel like they need to “act more like men”). Another potential consequence of a purely gender-blind ideology, which disregards some of the unique challenges women face, could be to blame women for their disadvantaged position. That is, gender-blindness can, while being empowering, also enhance “victim blaming” (see Kim et al., 2018).

### Recommendations for Future Research

One area that diversity ideologies research for both race-ethnicity and gender needs more work is the conceptual clarification of the ideological messages. As we discussed above scholars use a myriad ways to measure or manipulate different ideological messages. Some of the reviewed work clearly demonstrates that the specific elements of a diversity ideology message are consequential for how target groups respond to these. For the future, it remains important to clearly define the ideology in question, and even to test how slight differences in its focus (e.g., colorblindness that emphasizes an overarching group identity vs. individual uniqueness) influence women's and minorities responses.

Another area that needs more attention in future research is the study of intersectionality. Intersectionality research is concerned with the study of the impact of having multiple, often disadvantaged, identities (e.g., woman and minority) on individuals' experiences and behavior (Purdie-Vaughns and Eibach, 2008). From an intersectionality perspective, studying minority women as a separate group would provide unique insights because, given their multiple disadvantaged identities, this group's experiences may differ from both minority men and majority group women. That is, minority women may experience impediments as a consequence of both their gender and race-ethnicity, whereas minority men may primarily experience racial bias and majority group women gender bias. These more complex identity configurations may be especially relevant for diversity ideology research given the contrasting effects of gender and race-ethnicity focused ideologies as described above (e.g., Wilton et al., 2015; Martin and Phillips, 2017). Moreover, research on intersectional identity and stereotypes suggests that such research could provide insights that are specific for the experiences of distinct minority women groups. For example, given that the femininity stereotype applies much more strongly to some minority groups (e.g., Asian American) than others (e.g., African American; Galinsky et al., 2013), a gender aware ideology, which arguably emphasizes gender stereotypes may have substantially different consequences for women from either minority group. Further, beyond racial minority women, many individuals within groups differ in their identification, experiences, stigma-consciousness, as well as many other factors (Deaux et al., 1985; Bem, 1993; Pinel, 1999). As mentioned above, awareness and blindness ideologies have different effects based on a number of these factors. Therefore, it is important to understand not only broad level effects on racial minorities and women, but extend research to other factors that intersect with these identities.

The bulk of research on diversity ideologies focuses on racial minorities. Within this work, much of the research has examined the role of diversity ideologies in the U.S. context, focusing primarily on White- and African Americans, whose relations are often seen as hostile, contentious, and anxiety-ridden (Markus et al., 2000; Ryan et al., 2007). Thus, they may have unique effects, compared to interethnic relations involving different minority groups. Therefore, more work on how multiculturalism and colorblindness affect other ethnic groups such as Hispanics, East and South Asians, Middle Eastern, and Biracial individuals is needed. For example, individuals are more likely to endorse positive stereotypes about Asian Americans (the “model minority”; Wong et al., 1998). Although multiculturalism may heighten positive stereotypes about Asians, these stereotypes have pernicious and insidious effects, leading to feelings of marginalization, negative emotions, and decreased well-being and mental health (Sue et al., 2007; Siy and Cheryan, 2013; Czopp et al., 2015). Thus, it is imperative for research to go beyond targets who have historically hostile intergroup relations, to understand how multiculturalism affects many different ethnic groups.

Although research on diversity ideologies mainly focused on race, there is also increasing awareness for the role of diversity ideologies for women. As a result, this contribution also focused on these two groups' responses. However, racial-ethnic minorities and women are not the only potential demographic groups of interest. For instance, with the aging population, understanding the role diversity ideologies in the context of age diversity becomes a relevant question. To our knowledge, there is only a single study that examined the role of diversity ideologies in the context of age diversity. This work has demonstrated that organizational multi-age approach (i.e., a diversity aware ideology with a focus on recognition and celebration of age differences) is associated with both positive perceptions of older employees by others and older employees' reduced turnover intentions (Iweins et al., 2013). Diversity awareness can thus be beneficial for older employees (for similar arguments pertaining to broader age-inclusive HR practices see Boehm et al., 2014). For future work it is important to replicate these findings as well as to highlight their underlying reasons.

Besides diversity ideologies' impact on demographic groups based on visible characteristics (e.g., race, gender, age), it would also be valuable to examine these ideologies' effects on groups with invisible or concealable characteristics, such as sexual minorities. As a consequence of sexual minorities' emancipation in the last decades, gaining insight into the workplace experiences of these groups' has become a priority for many organizations. Moreover, academics have underlined the need for research examining the impact of identity aware vs. identity blind approaches for sexual minorities (Hebl et al., 2014). Future work should study the role of diversity ideologies on workplace experiences and outcomes of sexual minorities.

The current analysis on diversity ideologies in organizations focused primarily on the two dominant ideologies in the literature. It is important to note that more recently another promising ideological approach, polyculturalism, has been introduced. Polyculturalism focuses on “how cultures have interacted, influenced, and shared ideas and practices with each other throughout history, and how they continue to do so today” (Rosenthal and Levy, 2012, p. 2). By focusing on interconnectedness of and mutual influence between cultures, polyculturalism differs from multiculturalism as multiculturalism views cultures as distinct and separate entities. Research on polyculturalism has yielded important findings for diverse environments. For example, polyculturalism predicts intergroup contact and friendship (Rosenthal and Levy, 2016), an openness to cultural mixing (Cho et al., 2017) as well as lowered sexism (Rosenthal et al., 2014). Despite these promising findings, research on the impact of polyculturalism on workplace perceptions and outcomes is largely absent. Future work should examine the role the polyculturalist ideology plays in diverse workplace settings.

Further, much, if not most, research focuses on the ways in which diversity ideologies affect views of and behavior toward racial minorities and women; however, much less work has examined how these ideologies affect majority members views of themselves and behavior toward other majority members. For example, Plaut et al. (2011) find that Whites associate multiculturalism more with exclusion than inclusion. Martin and Phillips (2017) find that men who endorse gender-blindness are also more likely to identify with communal (i.e., gender-incongruent) traits. Thus, while understanding how diversity ideologies affect minority groups' self-perceptions is the primary focus of the current contribution, it is also important to understand how these ideologies influence dominant groups' self-perceptions.

As we discuss above, diversity ideologies, however, are not only organizational-level phenomena but can also refer individual level beliefs. That is, individual employees also differ in the extent to which they endorse diversity aware vs. diversity blind ideologies. Person-organization fit literature suggests that the (perceived) overlap between employee and organizational values is highly consequential for employee behavior (Kristof-Brown et al., 2005). Studying the interaction between individual and organizational level ideologies is an important avenue for future research to grasp the complexities of employee responses to diversity ideologies. Relatedly, future research can pay attention to potential “spill-over effects” between organizational initiatives (such as training) on ideologies held by individual employees. For example, it is possible that implicit bias trainings may make employees more “aware,” while policies such as performance-based reward may make them more “blind.” Although the current article zooms into diversity ideologies, interactions between diversity initiatives and employee ideological beliefs are a possibly fruitful avenue for future research.

Finally, we focused our review on organizational contexts, as these are where racial minorities and women are highly underrepresented (Catalyst, 2016; Fortune, 2017), and these contexts hold the most opportunity for power, influence, resources, and therefore equality between groups. Conducting more studies diversity ideologies on a national level would be valuable for the future, especially given that governments not only utilize these approaches but also countries differ in them (e.g., the U.S.' “melting-pot” vs. Canada's multiculturalism approach to diversity; Guimond et al., 2013). Indeed, work has found that above and beyond individual's endorsement of diversity ideologies, prejudice against Muslims is reduced when stronger multiculturalism policies are in effect (Guimond et al., 2013). For future work, it is imperative to illuminate the role of national ideologies on racial minorities' and women's perceptions and experiences.

## Conclusion

Research on diversity ideologies is relatively new but has generated some key insights in the last decades, especially in the context of racial and ethnic diversity. Emerging research on gender ideologies adds to this line of work and raises new theoretical questions and practical challenges that need to be addressed in the future. Overall, our review illustrates the important role of beliefs around diversity on the quality of intergroup relations focusing primarily on the target's perspective, with key implications for organizations and society.

## Author Contributions

All authors contributed to decisions with regards to initial focus and scope of the review. SG wrote the first draft of the manuscript. All authors contributed to manuscript revision, read, and approved the submitted version.

### Conflict of Interest Statement

The authors declare that the research was conducted in the absence of any commercial or financial relationships that could be construed as a potential conflict of interest.

## Footnotes

^1^Please note that we review prior work that has separately studied ideologies as contextual vs. personal variables. In the general discussion, we present recommendations for integrating these different levels of analysis in single studies in the future.

## References

[B1] Annis B. Merron K. (2014). Gender Intelligence: Breakthrough Strategies for Increasing Diversity and Improving Your Bottom Line. New York, NY: Harper Business.

[B2] Apfelbaum E. P. Norton M. I. Sommers S. R. (2012). Racial color blindness: emergence, practice, and implications. Curr. Dir. Psychol. Sci. 21, 205–209. 10.1177/0963721411434980

[B3] Apfelbaum E. P. Stephens N. M. Reagans R. E. (2016). Beyond one-size-fits-all:Tailoring diversity approaches to social groups. J. Pers. Soc. Psychol. 111, 547–566. 10.1037/pspi0000071 27428047

[B4] Arends-Tóth J. Van De Vijver F. J. R. (2003). Multiculturalism and acculturation: views of Dutch and Turkish-Dutch. Eur. J. Soc. Psychol. 33, 249–266. 10.1002/ejsp.143

[B5] Barreto M. Ellemers N. Piebinga L. Moya M. (2010). How nice of us and how dumb of me: The effect of exposure to benevolent sexism on women's task and relational self-descriptions. Sex Roles. 62, 532–544. 10.1007/s11199-009-9699-0

[B6] Barta T. Kleiner M. Neuman T. (2012). Is There a Payoff From Top Team Diversity? McKinsey Quarterly. Available online at: http://www.mckinsey.com/business-functions/organization/our-insights/is-there-a-payoff-from-top-team-diversity

[B7] Bastian B. Haslam N. (2006). Psychological essentialism and stereotype endorsement. J. Exp. Soc. Psychol. 42, 228–235. 10.1016/j.jesp.2005.03.003

[B8] Bem S. L. (1993). The Lenses of Gender: Transforming the Debate on Sexual Inequality. New Haven, CT: Yale University Press.

[B9] Boehm S. A. Kunze F. Bruch H. (2014). Spotlight on agediversity climate: the impact of Age inclusive HR practices on firm-level outcomes. Pers. Psychol. 67, 667–704. 10.1111/peps.12047

[B10] Bourguignon D. (2017). Diversity Policies, Prejudice, and Well-Being of Employees in Luxembourgish Organizations. Paper presented at the Diversity in Organizations Conference at Solvay Business School, (Brussels).

[B11] Castilla E. J. Benard S. (2010). The paradox of meritocracy in organizations. Adm. Sci. Q. 55, 543–676. 10.2189/asqu.2010.55.4.543

[B12] Catalyst (2016). Women CEOs of Sand P 500. Available online at: http://www.catalyst.org/knowledge/women-ceos-sp-500

[B13] Cho J. Morris M. W. Slepian M. L. Tadmor C. T. (2017). Choosing fusion: The effects of diversity ideologies on preference for culturally mixed experiences. J. Exp. Soc. Psychol. 69, 163–171. 10.1016/j.jesp.2016.06.013

[B14] Colby S. L. Ortman J. M. (2015). Projections of the Size and Composition of the U.S. Population: 2014 to 2060. 2015. Availale online at: https://www.census.gov/content/dam/Census/library/publications/2015/demo/p25-1143.pdf

[B15] Costa-Lopes R. Pereira C. R. Judd C. M. (2014). Categorisation salience and ingroup bias: The buffering role of a multicultural ideology. Int. J. Psychol. 49, 508–512. 10.1002/ijop.12044 25355674

[B16] Crosby F. J. Iyer A. Sincharoen S. (2006). Understanding affirmative action. Ann. Rev. Psychol. 57, 585–611. 10.1146/annurev.psych.57.102904.190029 16318608

[B17] Crosby J. R. King M. Savitsky K. (2014). The minority spotlight effect. Soc. Psychol. Pers. Sci. 5, 743–750. 10.1177/1948550614527625

[B18] Czopp A. M. (2008). When is a compliment not a compliment? Evaluating expressions of positive stereotypes. J. Exp. Soc. Psychol. 44, 413–420. 10.1016/j.jesp.2006.12.007

[B19] Czopp A. M. Kay A. C. Cheryan S. (2015). Positive stereotypes are pervasive and powerful. Pers. Psychol. Sci. 10, 451–463. 10.1177/1745691615588091 26177947

[B20] Deaux K. Winton W. Crowley M. Lewis L. L. (1985). Level of categorization and content of gender stereotypes. Soc. Cogn. 3, 145–167. 10.1521/soco.1985.3.2.145

[B21] Dover T. L. Major B. Kaiser C. R. (2016). Members of high-status groups are threatened by pro-diversity organizational messages. J. Exp. Soc. Psychol. 62, 58–67. 10.1016/j.jesp.2015.10.006

[B22] Dovidio J. F. Gaertner S. L. Saguy T. (2007). Another view of “we”: Majority and minority group perspectives on a common ingroup identity. Eur. Rev. Soc. Psychol. 18, 296–330. 10.1080/10463280701726132

[B23] Dovidio J. F. Saguy T. Gaertner S. L. (2010). Appreciating the role of the “individual mind” in diversity science: commonality, harmony, and social change. Psychol. Inquiry 21, 108–114. 10.1080/1047840X.2010.486071

[B24] Eagly A. H. (1997). Sex differences in social behavior: Comparing social role theory and evolutionary psychology. Am. Psychol. 50, 1380–1383. 10.1037/0003-066X.52.12.1380.b 9414607

[B25] Eagly A. H. Karau S. J. (2002). Role congruity theory of prejudice toward female leaders. Psychol. Rev. 109, 573–598 10.1037/0033-295X.109.3.573 12088246

[B26] Ely R. J. Thomas D. A. (2001). Cultural diversity at work: the effects of diversity perspectives on work group processes and outcomes. Adm. Sci. Q. 46, 229–273. 10.2307/2667087

[B27] Eurostat (2018). Immigration by Age Group, Sex and Citizenship. Available online at: http://appsso.eurostat.ec.europa.eu/nui/submitViewTableAction.do

[B28] Fiske S. T. Cuddy A. J. C. Glick P. Xu J. (2002). A model of (often mixed) stereotype content: competence and warmth respectively follow from perceived status and competition. J. Pers. Soc. Psychol. 82, 878–902. 10.1037/0022-3514.82.6.878 12051578

[B29] Fortune (2017). White Men Account for 72% of Corporate Leadership at 16 of the Fortune 500 Companies. Available online at: http://fortune.com/2017/06/09/white-men-senior-executives-fortune-500-companies-diversity-data/

[B30] Fryberg S. A. Stephens N. M. (2010). When the world is colorblind, American Indians are invisible. Psychol. Inquiry 21, 115–119. 10.1080/1047840X.2010.483847

[B31] Gaertner S. L. Dovidio J. F. (2014). Reducing Intergroup Bias: the Common Ingroup Identity Model. Philadelphia, PA: Psychology Press 10.4324/9781315804576

[B32] Galinsky A. D. Hall E. V. Cuddy A. J. (2013). Gendered races: Implications for interracial marriage, leadership selection, and athletic participation. Psychol. Sci. 24, 498–506. 10.1177/0956797612457783 23474830

[B33] Galinsky A. D. Todd A. R. Homan A. C. Phillips K. W. Apfelbaum E. P. Sasaki S. J.. (2015). Maximizing the gains and minimizing the pains of diversity. Pers. Psychol. Sci. 10, 742–748. 10.1177/1745691615598513 26581729

[B34] Glick P. Fiske S. T. (1996). The ambivalent sexism inventory: Differentiating hostile and benevolent sexism. J. Pers. Soc. Psychol. 70, 491–512. 10.1037/0022-3514.70.3.491

[B35] Government of Canada (2018). Multiculturalism. Available online at: https://www.canada.ca/en/services/culture/canadian-identity-society/multiculturalism.html

[B36] Guimond S. Crisp R. J. De Oliveira P. Kamiejski R. Kteily N. Kuepper B.. (2013). Diversity policy, social dominance, and intergroup relations: Predicting prejudice in changing social and political contexts. J. Pers. Soc. Psychol. 104, 941–958. 10.1037/a0032069 23527848

[B37] Gündemir S. Dovidio J. F. Homan A. C. De Dreu C. K. W. (2017a). The impact of organizational diversity policies on minority employees' leadership self-perceptions and goals. J. Leadersh. Organ. Stud. 24, 172–188. 10.1177/1548051816662615

[B38] Gündemir S. Galinsky A. D. (2018). Multicolored blindfolds: how organizational multiculturalism can conceal racial discrimination and delegitimize racial discrimination claims. Soc. Psychol. Pers. Sci. 9, 825–834. 10.1177/1948550617726830

[B39] Gündemir S. Homan A. C. de Dreu C. K. W. van Vugt M. (2014). Think leader, think white? Capturing and weakening an implicit pro-white leadership bias. PLoS ONE 9:e83915. 10.1371/journal.pone.0083915 24416181 PMC3885528

[B40] Gündemir S. Homan A. C. Usova A. Galinsky A. D. (2017b). Multicultural meritocracy: the synergistic benefits of valuing both diversity and merit. J. Exp. Soc. Psychol. 73, 34–41. 10.1016/j.jesp.2017.06.002

[B41] Gutiérrez A. S. Unzueta M. M. (2010). The effect of inter- ethnic ideologies on the likability of stereotypic vs. counter- stereotypic minority targets. J. Exp. Soc. Psychol. 46, 775–784. 10.1016/j.jesp.2010.03.010

[B42] Hahn A. Banchefsky S. Park B. Judd C. M. (2015). Measuring intergroup ideologies: positive and negative aspects of emphasizing versus looking beyond group differences. Pers. Soc. Psychol. Bull. 41, 1646–1664. 10.1177/0146167215607351 26453053

[B43] Hahn A. Judd C. M. Park B. (2010). Thinking about group differences: ideologies and national identities. Psychol. Inquiry 21, 120–126. 10.1080/1047840X.2010.483997

[B44] Hebl M. R. Martinez L. R. Skorinko J. L. Barron L. G. King E. B. (2014). How diversity ideologies influence LGBT employees: to be or not to be; and to see or not to see, in Diversity ideologies in organizations, eds, Thomas K. M. Plaut V. Tran M. (New York, NY: Taylor and Francis), 151–176.

[B45] Hehman E. Gaertner S. L. Dovidio J. F. Mania E. W. Guerra R. Wilson D. C.. (2012). Group status drives majority and minority integration preferences. Psychol. Sci. 23, 46–52. 10.1177/0956797611423547 22173737

[B46] Heilman M. E. Simon M. C. Repper D. P. (1987). Intentionally favored, unintentionally harmed? Impact of sex-based preferential selection on self-perceptions and self-evaluations. J. Appl. Psychol. 72, 62–68. 10.1037/0021-9010.72.1.62

[B47] Hideg I. Ferris D. L. (2016). The compassionate sexist? How benevolent sexism promotes and undermines gender equality in the workplace. J. Pers. Soc. Psychol. 111, 706–727. 10.1037/pspi0000072 27414234

[B48] Holoien D. S. Shelton J. N. (2012). You deplete me: the cognitive costs of colorblindness on ethnic minorities. J. Exp. Soc. Psychol. 48, 562–565. 10.1016/j.jesp.2011.09.010

[B49] Homan A. C. van Knippenberg D. Van Kleef G. A. De Dreu C. K. W. (2007). Bridging faultlines by valuing diversity: the effects of diversity beliefs on information elaboration and performance in diverse work groups. J. Appl. Psychol. 92, 1189–1199. 10.1037/0021-9010.92.5.1189 17845079

[B50] Ito T. A. Urland G. R. (2003). Race and gender on the brain: electrocortical measures of attention to the race and gender of multiply categorizable individuals. J. Pers. Soc. Psychol. 85, 616–626. 10.1037/0022-3514.85.4.616 14561116

[B51] Iweins C. Desmette D. Yzerbyt V. Stinglhamber F. (2013). Ageism at work: The impact of intergenerational contact and organizational multi-age perspective. Eur. J. Work Organ. Psychol. 22, 331–346. 10.1080/1359432X.2012.748656

[B52] Jackson S. E. Joshi A. Erhardt N. L. (2003). Recent research on team and organizational diversity: SWOT analysis and implications. J. Manag. 29, 801–830. 10.1016/S0149-2063(03)00080-1

[B53] Jansen W. S. Vos M. W. Otten S. Podsiadlowski A. van der Zee K. I. (2016). Colorblind or colorful? How diversity approaches affect cultural majority and minority employees. J. Appl. Soc. Psychol. 46, 81–93. 10.1111/jasp.12332

[B54] Joshi Y. (2014). The trouble with inclusion. Virginia J. Soc. Policy Law. 21, 207–265.

[B55] Kaiser C. R. Major B. Jurcevic I. Dover T. L. Brady L. M. Shapiro J. R. (2013). Presumed fair: Ironic effects of organizational diversity structures. J. Pers. Soc. Psychol. 104, 504–519. 10.1037/a0030838 23163748

[B56] Kalev A. Dobbin F. Kelly E. (2006). Best practices or best guesses? Diversity management and the remediation of inequality. Am. Sociol. Rev. 71, 589–617. 10.1177/000312240607100404

[B57] Kelly E. Dobbin F. (1998). How affirmative action became diversity management: employer response to antidiscrimination law. 1961 to 1996. Am. Behav. Sci. 41, 960–984. 10.1177/0002764298041007008

[B58] Kim J.Y. Fitzsimons G. M. Kay A. C. (2018). Lean in messages increase attributions of women's responsibility for gender inequality. J. Pers. Soc. Psychol. 115, 974–1001. 10.1037/pspa0000129 30550322

[B59] Koenig A. Richeson J. (2010). The contextual endorsement of sexblind versus sexaware ideologies. Soc. Psychol. 41, 186–191 10.1027/1864-9335/a000026

[B60] Konrad A. M. Linnehan F. (1995). Formalized HRM structures: coordinating equal employment opportunity or concealing organizational practices?. Acad. Manag. J. 38, 787–820. 10.5465/256746

[B61] Krawcheck S. (2017). Own it: The Power of Women at Work. New York, NY: Crown Business.

[B62] Kristof-Brown A. L. Zimmerman R. D. Johnson E. C. (2005). Consequences of individuals' fit at work: a meta-analysis of person-job, person-organization, person-group, and person-supervisor fit. Pers. Psychol. 58, 281–342. 10.1111/j.1744-6570.2005.00672.x

[B63] Leslie L. M. Mayer D. M. Kravitz D. A. (2014). The stigma of affirmative action: a stereotyping- based theory and meta-analytic test of the consequences for performance. Acad. Manag. J. 57, 964–989. 10.5465/amj.2011.0940

[B64] Levin S. Matthews M. Guimond S. Sidanius J. Pratto F. Kteily N. (2012). Assimilation, multiculturalism, and colorblindness: mediated and moderated relationships between social dominance orientation and prejudice. J. Exp. Soc. Psychol. 48, 207–212. 10.1016/j.jesp.2011.06.019

[B65] Malicke J. (2013). Gender Ideology and Evaluations of a Male Versus Female Target: Effects of Emphasizing Versus Downplaying Gender Differences. Unpublished Master's Thesis, University of Colorado Boulder.

[B66] Markus H. R. Steele C. M. Steele D. M. (2000). Colorblindness as a barrier to inclusion: Assimilation and nonimmigrant minorities. Daedalus. 129, 233–259. Available online at: https://www.jstor.org/stable/20027672

[B67] Martin A. E. (2018). The Divergent Effects of Diversity Ideologies for Race and Gender Relations (No. 10789688). ProQuest Dissertations and Theses Global (2034426489).

[B68] Martin A. E. Gündemir S. Phillips K. W. Homan A. C. (2018). Women's Responses to Gender-Aware and Gender-Blind Organizational Approaches: The Moderating Role of Career and Family Orientation. Working Paper, Columbia Business School, New York, NY.

[B69] Martin A. E. Phillips K. W. (2017). What “blindness” to gender differences helps women see and do: Implications for confidence, agency, and action in male-dominated environments. Organ. Behav. Hum. Dec. Proc. 142, 28–44. 10.1016/j.obhdp.2017.07.004

[B70] Martin A. E. Phillips K. W. (2019). Blind to bias: the benefits of gender-blindness for STEM Stereotyping. J. Exp. Soc. Psychol. 10.1016/j.jesp.2018.11.003

[B71] Martin A. E. Phillips K. W. Sasaki S. J. (2016). The Benefits of Gender-Blindness for Men's Bias Towards and Inclusion of Women. Paper presented at Academy of Management Annual Meeting, Anaheim, CA 10.5465/ambpp.2016.14581abstract

[B72] Martin C. L. Parker S. (1995). Folk theories about sex and race differences. Pers. Soc. Psychol. Bull. 21, 45–57. 10.1177/0146167295211006

[B73] Meeussen L. Otten S. Phalet K. (2014). Managing diversity: How leaders' multiculturalism and colorblindness affect work group functioning. Group Processes and Intergroup Relations. 17, 629–644. 10.1177/1368430214525809

[B74] Morrison K. R. Chung A. H. (2011). “White” or “European American”? Self-identifying labels influence majority group members' interethnic attitudes. J. Exp. Soc. Psychol. 47, 165-170. 10.1016/j.jesp.2010.07.019

[B75] MTV Bias Survey (2014). MTV Survey on Millennials and Race: Executive Summary: MTV Strategic Insights. New York, NY. Available online at: http://d1fqdnmgwphrky.cloudfront.net/studies/000/000/001/DBR_MTV_Bias_Survey_Executive_Summary.pdf?1398858309

[B76] Ospina S. Foldy E. (2009). A critical review of race and ethnicity in the leadership literature: Surfacing context, power and the collective dimensions of leadership. Leadership Q. 20, 876–896. 10.1016/j.leaqua.2009.09.005

[B77] Page S. E. (2007). The Difference: How the Power of Diversity Creates Better Groups, Firms, Schools, and Societies. Princeton, NJ: Princeton University Press.

[B78] Park B. Judd C. M. (2005). Rethinking the link between categorization and prejudice within the social cognition perspective. Pers. Soc. Psychol. Rev. 9, 108–130. 10.1207/s15327957pspr0902_2 15869378

[B79] Peery D. (2011). The colorblind ideal in a race-conscious reality: the case for a new legal ideal for race relations. Northwest. J. Law Soc. Policy. 6, 473–495. Available online at: https://scholarlycommons.law.northwestern.edu/cgi/viewcontent.cgi?article=1070&context=njlsp

[B80] Pelled L. H. Eisenhardt K. M. Xin K. R. (1999). Exploring the black box: an analysis of work group diversity, conflict, and performance. Adm. Sci. Q. 44, 1–28. 10.2307/2667029

[B81] Phouthonephackdy T. (2016). Diversity Climate Perceptions and Employee Turnover Intentions: The Importance of Racial Group Identification (Master's thesis), University of Waterloo.

[B82] Pinel E. C. (1999). Stigma consciousness: the psychological legacy of social stereotypes. J. Pers. Soc. Psychol. 76, 114–128. 10.1037/0022-3514.76.1.114 9972557

[B83] Plaut V. C. (2010). Diversity science: why and how difference makes a difference. Psychol. Inquiry 21, 77–99. 10.1080/10478401003676501

[B84] Plaut V. C. Garnett F. G. Buffardi L. E. Sanchez-Burks J. (2011). “What about me?” Perceptions 18 of exclusion and Whites' reactions to multiculturalism. J. Pers. Soc. Psychol. 101, 337–353. 10.1037/a0022832 21534702

[B85] Plaut V. C. Thomas K. M. Goren M. J. (2009). Is multiculturalism or colorblindness better for minorities? Psychol. Sci. 20, 444–446. 10.1111/j.1467-9280.2009.02318.x 19399972

[B86] Plaut V. C. Thomas K. M. Hurd K. Romano C. A. (2018). Do color blindness and multiculturalism remedy or foster discrimination and racism?. Curr. Dir. Psychol. Sci. 27, 200–206. 10.1177/0963721418766068

[B87] Purdie-Vaughns V. Eibach R. P. (2008). Intersectional invisibility: The distinctive advantages and disadvantages of multiple subordinate-group identities. Sex Roles. 59, 377–391. 10.1007/s11199-008-9424-4

[B88] Purdie-Vaughns V. Steele C. M. Davies P. G. Ditlmann R. Crosby J. R. (2008). Social identity contingencies: how diversity cues signal threat or safety for African Americans in mainstream institutions. J. Pers. Soc. Psychol. 94, 615–630. 10.1037/0022-3514.94.4.615 18361675

[B89] Purdie-Vaughns V. Walton G. M. (2011). Is multiculturalism bad for African Americans? Redefining inclusion through the lens of identity safety, in Moving Beyond Prejudice Reduction: Pathways to Positive Intergroup Relations, eds, Tropp L. R. Mallett R. K. (Washington, DC: American Psychological Association), 159–177.

[B90] Rattan A. Ambady N. (2013). Diversity ideologies and intergroup relations: an examination of colorblindness and multiculturalism. Eur. J. Soc. Psychol. 43, 12–21. 10.1002/ejsp.1892

[B91] Rosch E. Lloyd B. B. (1978). Cognition and Categorization. Vol. 1 Hillsdale, NJ: Lawrence Erlbaum Associates.

[B92] Rosenthal L. Levy S. R. (2010). The colorblind, multicultural, and polycultural ideological approaches to improving intergroup attitudes and relations. Soc. Issues Policy Rev. 4, 215–246. 10.1111/j.1751-2409.2010.01022.x

[B93] Rosenthal L. Levy S. R. (2012). The relation between polyculturalism and intergroup attitudes among racially and ethnically diverse adults. Cult. Divers. Ethnic Minor. Psychol. 18, 1–16. 10.1037/a0026490 22250894

[B94] Rosenthal L. Levy S. R. (2016). Endorsement of polyculturalism predicts increased positive intergroup contact and friendship across the beginning of college. J. Social Issues 72, 472–488. 10.1111/josi.12177

[B95] Rosenthal L. Levy S. R. Militano M. (2014). Polyculturalism and sexist attitudes: Believing cultures are dynamic relates to lower sexism. Psychol. Wom. Q. 38, 519–534. 10.1177/0361684313510152 25530662 PMC4266561

[B96] Ryan C. S. Casas J. F. Thompson B. K. (2010). Interethnic ideology, intergroup perceptions, and cultural orientation. J. Soc. Issues 66, 29–44. 10.1111/j.1540-4560.2009.01631.x

[B97] Ryan C. S. Hunt J. S. Weible J. A. Peterson C. R. Casas J. F. (2007). Multicultural and colorblind ideology, stereotypes, and ethnocentrism among Black and White Americans. Group Proc. Interg. Rel. 10, 617–637. 10.1177/1368430207084105

[B98] Saguy T. Tausch N. Dovidio J. F. Pratto F. (2009). The irony of harmony: intergroup contact can produce false expectations for equality. Psychol. Sci. 20, 114–121. 10.1111/j.1467-9280.2008.02261.x 19152543

[B99] Sasaki S. J. Vorauer J. D. (2013). Ignoring versus exploring differences between groups: Effects of salient color-blindness and multiculturalism on intergroup attitudes and behavior. Soc. Pers. Psychol. Compass. 7, 246–259. 10.1111/spc3.12021

[B100] Schein E. (1992). Organizational Culture and Leadership. San Francisco, CA: Jossey-Bass.

[B101] Schofield J. W. (2001). The colorblind perspective in school: causes and consequences, in Multicultural Education. Issues and Perspectives, eds Banks J. A. McGeeBanks C. A. (New York, NY: Wiley), 247–267.

[B102] Shnabel N. Bar-Anan Y. Kende A. Bareket O. Lazar Y. (2016). Help to perpetuate traditional gender roles: Benevolent sexism increases engagement in dependency-oriented cross-gender helping. J. Pers. Soc. Psychol. 110, 55–75. 10.1037/pspi0000037 26461798

[B103] Siy J. O. Cheryan S. (2013). When compliments fail to flatter: American individualism and responses to positive stereotypes. J. Pers. Soc. Psychol. 104, 87–102 10.1037/a0030183 23025500

[B104] Stevens F. G. Plaut V. C. Sanchez-Burks J. (2008). Unlocking the benefits of diversity: All- inclusive multiculturalism and positive organizational change. J. Appl. Behav. Sci. 44, 116–133. 10.1177/0021886308314460

[B105] Sue D. W. Capodilupo C. M. Torino G. C. Bucceri J. M. Holder A. Nadal K. L. (2007). Racial micro-aggressions in everyday life: implications for clinical practice. Am. Psychol. 62, 271–286. 10.1037/0003-066X.62.4.271 17516773

[B106] Todd A. R. Galinsky A. D. (2012). The reciprocal link between multiculturalism and perspective-taking: how ideological and self-regulatory approaches to managing diversity reinforce each other. J. Exp. Soc. Psychol. 48, 1394–1398. 10.1016/j.jesp.2012.07.007

[B107] Van Knippenberg D. De Dreu C. K. W. Homan A. C. (2004). Work group diversity and group performance: an integrative model and research agenda. J. Appl. Psychol. 89, 1008–1022. 10.1037/0021-9010.89.6.1008 15584838

[B108] Van Laar C. Derks B. Ellemers N. (2013). Motivation for education and work in young Muslim women: the importance of value for ingroup domains. Basic Appl. Soc. Psychol. 35, 64–74. 10.1080/01973533.2012.746609

[B109] Verkuyten M. (2005). Ethnic group identification and group evaluation among minority and majority groups: testing the multiculturalism hypothesis. J. Pers. Soc. Psychol. 88, 121–138. 10.1037/0022-3514.88.1.121 15631579

[B110] Verkuyten M. (2009). Self-esteem and multiculturalism: an examination among ethnic minority and majority groups in the Netherlands. J. Res. Pers. 43, 419–427. 10.1016/j.jrp.2009.01.013

[B111] Verkuyten M. (2010). Assimilation ideology and situational well-being among ethnic minority members. J. Exp. Soc. Psychol. 46, 269–275. 10.1016/j.jesp.2009.11.007

[B112] Vescio T. K. Gervais S. J. Snyder M. Hoover A. (2005). Power and the creation of patronizing environments: the stereotype-based behaviours or the powerful and their effects on female performance in masculine domains. J. Pers. Soc. Psychol. 88, 658–672. 10.1037/0022-3514.88.4.658 15796666

[B113] Vorauer J. D. Gagnon A. Sasaki S. J. (2009). Salient intergroup ideology and intergroup interaction. Psychol. Sci. 20, 838–845. 10.1111/j.1467-9280.2009.02369.x 19493325

[B114] Wilder D. A. (1984). Prediction of belief homogeneity and similarity following social categorization. Br. J. Soc. Psychol. 23, 323–333 10.1111/j.2044-8309.1984.tb00648.x

[B115] Wilton L. S. Apfelbaum E. P. Good J. J. (2018). Valuing differences and reinforcing them: multiculturalism increases race essentialism. Soc. Psychol. Pers. Sci. 10.1177/1948550618780728

[B116] Wilton L. S. Good J. J. Moss-Racusin C. A. Sanchez D. T. (2015). Communicating more than diversity: The effect of institutional diversity statements on expectations and performance as a function of race and gender. Cult. Div. Ethnic Min. Psychol. 21, 315–325. 10.1037/a0037883 25313429

[B117] Wolsko C. Park B. Judd C. M. (2006). Considering the tower of Babel: Correlates of assimilation and multiculturalism among ethnic minority and majority groups in the United States. Soc. Just. Res. 19, 277–306. 10.1007/s11211-006-0014-8

[B118] Wolsko C. Park B. Judd C. M. Wittenbrink B. (2000). Framing interethnic ideology: E?ects of multicultural and color-blind perspectives on judgments of groups and individuals. J. Pers. Soc. Psychol. 78, 635–654. 10.1037/0022-3514.78.4.635 10794371

[B119] Wong P. Lai C. F. Nagasawa R. Lin T. (1998). Asian Americans as a model minority: Self-perceptions and perceptions by other racial groups. Sociol. Pers. 41, 95–118. 10.2307/1389355

[B120] Zou L. Cheryan S. (2015). When whites' attempts to be multicultural backfire in intergroup interactions. Soc. Pers. Psychol. Compass 9, 581–592. 10.1111/spc3.12203

